# A search for genetic markers associated with egg production in the ostrich (*Struthio camelus*)

**DOI:** 10.1007/s11033-012-1632-x

**Published:** 2012-04-28

**Authors:** M. Kawka, J. O. Horbańczuk, K. Jaszczak, M. Pierzchała, R. G. Cooper

**Affiliations:** 1 Institute of Genetics and Animal Breeding of the Polish Academy of Sciences, Jastrzębiec, Poland; 2 Eurohouse, Dog Kennel Lane, Walsall, West Midlands, UK

**Keywords:** Microsatellite markers, Egg production, Ostrich, *Struthio camelus*

## Abstract

The aim of the current study was to search for genetic markers, microsatellite *loci* associated with laying performance in ostriches. The material consisted of two groups of ostrich hens characterized by high or low laying performance (over 75 and less than 25 eggs per season, respectively). The investigation covered 30 microsatellite *loci* characteristic for the ostrich (the CAU group) and led to identification of significant differences in allele and genotype frequencies between the two groups of hens considered. Out of a total of 30 microsatellite *loci* examined, 28 showed different alleles in relation to analyzed performance groups. In hens of high laying performance (HP group, n = 12), specific alleles occurred in 23 microsatellite *loci* (40 alleles of 243 identified), while in those of low egg production (LP group, n = 12), they occurred in 22 (51 alleles of 243 identified). The results indicate the usefulness of the microsatellite *loci* as the potential genetic markers associated with laying performance that can be applied for genetic improvement of ostrich flocks.

## Introduction

Ostriches provide dietetic meat, valuable skin, feathers and eggs [3, 4, 27] that make them important alternative livestock in many parts of the world [5–7, 13, 22, 23]. However, one of the basic reasons for the hindered development of this new agricultural activity is its low reproduction rate [2, 14, 15, 25] and high housing costs. It is more profitable to keep one hen which produces 60–80 eggs per season than two hens with half of that egg production [11]. So it becomes necessary to obtain higher genetic progress of the production in laying ostrich hens. Due to the development of molecular methods, e.g. microsatellite sequences, new opportunities for genetic improvement of ostrich flocks have emerged in the last decades [8, 12, 16–19]. Microsatellite sequences are widely used as genetic markers, because they occur in the genome frequently, are evenly-distributed and show wide inter-individual variation and a high rate of heterozygosity [9, 17, 20, 21, 26]. Facing the above, the aim of the study was to identify specific genetic markers—microsatellite alleles—related to the laying performance in ostriches.

## Material and methods

The material consisted of 24 unrelated African Black ostrich hens kept in breeding pairs or trios at the Stypułów farm, Poland, which maintains the birds under conditions compliant with EU recommendations by the Committee of the European Convention for the Protection of Animals Kept for Farming Purposes (T-AP)—Draft Recommendation Concerning Ratites (Ostriches, Emus and Rheas). The study included the collection of non-invasive material only (feathers) which did not require the approval of an Ethics Committee. The Stypułów farm is under official scientific supervision of the Institute of Genetics and Animal Breeding of the Polish Academy of Sciences (official letter of agreement signed in 2002).

Two groups of hens in their third laying season (12 per group) were randomly completed according to maximum or minimum values of laying performance: group HP (high productivity)—with a total egg production of minimum 75 eggs per hen per season (mean of 78.92; SD = 5.00) and group LP (low productivity)—where egg production did not exceed 25 eggs per hen per season (mean 18.75; SD = 3.89).

Ostrich genomic DNA was isolated from feathers (non-invasive methods) using Dneasy Tissue KIT 250 (QUIAGEN). Each sample was examined both spectrophotometrically and electrophoretically. An analysis of 30 microsatellite *loci* characteristic of ostrich [28], derived from the CAU (China Agricultural University) group was performed. One of the primer pairs has been labeled with one of the four dyes—6-FAM, VIC, NED, PET. The amplification of selected microsatellite *loci* was performed using a thermal cycler PTC-200 Engine (MJ Research). The PCR was carried out in a total volume of 10 ml comprising 10 ng of template DNA, 0.5 mM of each nucleotide, 100 pmol of each primer, 1.5 mM MgCl2, 50 mM KCL, 10 mM Tris–HCL, 0.01 % Tryton X-100 and 0.5 units of DNA polymerase (POLGEN). For all tested microsatellite *loci* determined experimentally the thermal profile and the number of cycles was noted. The fluorescent PCR products were separated by electrophoresis using the four-capillary genetic analyzer (Applied Biosystems 3130) and the computer software (GeneScan). The results were visualized and the genotyping completed with GeneScan 2.1. In addition, the computer program GeneMapper (Applied Biosystems) was used to determine the allele size for the individual markers automatically.

The computer program GENPOP, version 4.1 [24] was used to determine: heterozygosity and polymorphism information content (PIC)—for evaluation the genetic variability and deviations from Hardy–Weinberg equilibrium. Expected heterozygosity (HETexp) was calculated from Hardy–Weinberg assumptions for each *locus* Formula. Deviations from Hardy–Weinberg equilibrium (HWE) [30] were tested by the Chi-squared test.

## Results and discussion

A preliminary study on the identification of genetic markers associated with the egg production of ostriches has earlier been conducted by Kawka et al. [18], but based mainly on the analysis of DNA fingerprinting including the genetic linkage between minisatellite DNA markers and quantitative trait (egg production). Methods based on minisatellite DNA markers did not distinguish bands specific for the high or low performance groups of hens. The results allowed to conclude neither about the potential linkage between alleles represented by specific hybridization bands or *loci* of genes, thereof coding for the control of egg production. It should be emphasized that the present study was based on microsatellite *loci* characteristic for the ostrich since it provides more detailed information and therefore is widely used in linkage mapping of farm animals QTLs. Analysis of the polymorphism of these *loci* led to the identification of alleles and *loci* differing between two groups of ostrich hens—with the high and low laying production.

Table 1 shows characteristics of ostrich groups with high (HP) and low (LP) laying performance, i.e*.* heterozygosity expected (H_e_), heterozygosity observed (H_o_), PIC index, genetic differentiation and deviations from Hardy–Weinberg equilibrium. Mean heterozygosity for 30 analyzed markers were similar in HP and LP groups. The H_o_ ranged from 0.25 to 1.00 (LP group) and from 0.17 to 1.00 (HP group). In turn, the values of (H_e_) estimated for population analyzed, ranged from 0.41 to 0.94 (LP) and from 0.50 to 0.93 (HP). Both mean values (H_o_ and H_e_) occurred relatively high (over 0.8) what indicates the high genetic variability of the population in question. Kawka et al. [17], analyzing the genetic variability within and among 3 ostrich breeds reported a mean observed and expected heterozygosity ranging from 0.463 to 0.663 and from 0.481 to 0.679, respectively. Kimwele and Graves [19] showed, that the H_e_ for an ostrich populations living in wild and kept on farms in Kenya, ranged from 0.40 to 0.79. In turn, Hammond et al. [10] in emu populations kept on farms in Australia reported this ratio to vary from 0.44 to 1.

**Table 1:** Heterozygosites (H_e_, H_o_), PIC, genic and genotypic linkage disequilibrium, and probability of deviation from Hardy–Weinberg equilibrium using Weir and Cockerham [30] for microsatellite *loci* between HP and LP groups of ostrich hens

*locus*	PIC			Het-o			Het-e			Genic differentiation (exact G test)	Genotypic differentiation (exact G test)	Hardy–Weinberg prob. test
	LP	HP	Overall	LP	HP	Overall	LP	HP	Overall	*p* value	*p* value	*p* value
CAU1	0.83	0.82	0.84	1.00	0.92	0.96	0.92	0.92	0.89	0.63	0.57	0.63
CAU3	0.78	0.68	0.74	1.00	1.00	1.00	0.88	0.80	0.81	0.38	0.26	0.11
CAU7	0.71	0.69	0.72	0.83	0.92	0.88	0.80	0.78	0.78	0.04	0.04	0.02
CAU11	0.78	0.79	0.80	0.83	0.92	0.88	0.88	0.89	0.86	0.38	0.33	0.07
CAU14	0.67	0.78	0.75	0.83	0.92	0.88	0.78	0.88	0.82	0.11	0.08	0.24
CAU16	0.77	0.76	0.78	0.92	1.00	0.96	0.86	0.86	0.84	0.85	0.80	0.26
CAU17	0.83	0.79	0.82	0.83	0.92	0.88	0.92	0.89	0.87	0.96	0.94	0.02
CAU22	0.65	0.64	0.65	1.00	1.00	1.00	0.77	0.75	0.73	1.00	1.00	0.01
CAU23	0.69	0.70	0.72	0.92	1.00	0.96	0.79	0.80	0.78	0.14	0.08	0.02
CAU25	0.68	0.70	0.71	1.00	1.00	1.00	0.80	0.81	0.78	0.48	0.36	0.00
CAU30	0.79	0.83	0.85	1.00	1.00	1.00	0.89	0.93	0.90	0.10	0.07	0.11
CAU32	0.83	0.76	0.83	0.42	0.17	0.29	0.92	0.86	0.88	0.03	0.30	0.00
CAU34	0.65	0.57	0.61	0.83	0.83	0.83	0.76	0.69	0.70	0.95	0.94	0.25
CAU40	0.76	0.74	0.76	1.00	1.00	1.00	0.86	0.84	0.82	0.46	0.35	0.28
CAU42	0.76	0.74	0.77	0.67	0.58	0.63	0.86	0.84	0.83	0.08	0.21	0.01
CAU43	0.75	0.75	0.75	0.92	1.00	0.96	0.85	0.85	0.82	0.97	0.96	0.80
CAU44	0.54	0.59	0.56	0.92	0.92	0.92	0.67	0.72	0.66	0.93	0.87	0.02
CAU57	0.61	0.61	0.63	0.25	0.67	0.46	0.71	0.70	0.69	0.13	0.27	0.01
CAU64	0.79	0.74	0.77	1.00	1.00	1.00	0.88	0.84	0.83	0.95	0.93	0.01
CAU65	0.75	0.77	0.77	0.83	0.83	0.83	0.86	0.87	0.83	0.84	0.83	0.48
CAU68	0.61	0.73	0.72	0.83	1.00	0.92	0.70	0.84	0.78	0.06	0.03	0.65
CAU69	0.76	0.76	0.78	1.00	1.00	1.00	0.86	0.86	0.84	0.21	0.13	0.27
CAU75	0.80	0.81	0.81	0.92	1.00	0.96	0.89	0.90	0.87	0.87	0.79	0.00
CAU76	0.85	0.81	0.85	0.92	1.00	0.96	0.94	0.91	0.90	0.36	0.34	0.03
CAU78	0.30	0.41	0.37	0.50	0.50	0.50	0.41	0.50	0.44	0.22	0.18	0.62
CAU83	0.81	0.71	0.79	0.67	0.58	0.63	0.91	0.81	0.85	0.17	0.43	0.00
CAU84	0.71	0.80	0.77	0.92	0.83	0.88	0.81	0.89	0.84	0.46	0.49	0.12
CAU85	0.89	0.84	0.90	1.00	1.00	1.00	0.98	0.93	0.95	0.04	0.04	0.02
CAU97	0.68	0.51	0.62	0.75	0.42	0.58	0.79	0.61	0.69	0.41	0.42	0.35
CAU98	0.76	0.81	0.80	1.00	1.00	1.00	0.86	0.91	0.86	0.39	0.29	0.23
Pooled	0.73	0.72	0.74	0.85	0.86	0.86	0.83	0.82	0.80	0.14	0.13	<0.005

As regards the PIC, the highest value of which (more than 0.7) was observed for 20 *loci* in LP and for 22 *loci* in HP group. The lowest values of the PIC (0.30 and 0.41) were recorded for *locus* CAU78 in LP and HP group, respectively (Table 1). Earlier Kawka et al. [17] reported the PIC in ostriches to range from 0.117 to 0.786. Almost all the microsatellite markers selected for the current analysis were characterized either by a high heterozygosity or high PIC values.

Generally it can be assumed that the studied ostrich population remained in Hardy–Weinberg (HWE) equilibrium (Table 1). However, several *loci* showed significant (*p* < 0.05) deviations from HWE (CAU22, CAU32, CAU42, CAU75, CAU83 and CAU84 in HP CAU25, CAU32, CAU57 and CAU83 in LP group). The further wider analysis would prove, whether *loci* showing such disequilibrium between observed and expected genotypes could be associated with laying performance in ostrich. The more precise estimation using genic and genotypic differentiation approach of GENEPOP showed significant differences of allele and genotype frequencies of individual *loci* between the two groups of layers (HP and LP): CAU7,CAU32, CAU 68, CAU85 (Table 1). However, overall analysis for all 30 *loci* together did not show significant differences between groups: Chi-square = 72.51 (df = 60), *p* value = 0.12. Moreover, out of a total of 30 microsatellite *loci* examined, 28 showed different alleles for both groups. Two microsatellite *loci* (CAU43 and CAU68) had no specific alleles in any of ostrich groups. In a total pool of 243 microsatellite alleles, 152 (62.5 %) were common for the two production groups. The most common alleles were observed at *locus* CAU17 (8 of 10 identified alleles) and CAU16, CAU43, CAU64 and CAU75-7 common alleles. In the *locus* CAU7, out of the total number of 12 alleles, only 3 were common for the studied groups of hens. Ninety one (over 37 %) microsatellite alleles from a total pool of alleles occurring in the genome of the two analyzed ostrich groups can be considered as specific for the group. Of these alleles, 40 (16.4 %) were typical for HP and 51 (20.9 %) for LP. The most of specific alleles occurred at the *locus* CAU7 (9 of the 12 identified) and CAU85 (9 of the 15 identified) (Table 2). Alleles specific for HP hens were identified at 23, while for LP hens—at 22 microsatellite *loci*. The most specific alleles for HP hens were identified at *loci* CAU7 and CAU85—4 alleles. Thirteen microsatellite *loci* were characterized by only one specific allele for this group of hens (Table 2). However, in the case of LP hens, the most specific alleles were observed at *loci* CAU7, CAU32 and CAU85—5 alleles. The one characteristic allele for these hens occurred in 7 analyzed microsatellite markers.

**Table 2:** Common and specific alleles for two analyzed groups of ostrich hens

*locus*	Alleles common for two groups of ostrich	Allele specific for the group	
		Hens with high productivity	Hens with low productivity
CAU1	84,86,90,94,96,104	88	92,98,100
CAU3	111,115,117,119	–	113,121
CAU7	185,187,205	189,195,207,209	183,191,197,203,211
CAU11	104,106,110,112, 114,118	–	98,100
CAU14	146,148,150,152	138,140,144	–
CAU16	188,190,192,194, 200,204,206	198	186
CAU17	160,162,164,166, 168,170,176,178	174	180
CAU22	142,144,146,150,152	–	148
CAU23	167,169,177,191	171,185	181,183,189,195
CAU25	199,201,203,205,207	197	–
CAU30	115,125,127,129,131,135	119,123,137	117,133
CAU32	179,183,185,189	197,199	187,191,193,203,205
CAU34	198,200,202,204	–	192,196
CAU40	142,144,146,148	150	140,152
CAU42	192,200,202,204,206	196,198	184,194
CAU43	209,211,213,215,217,219,221	–	–
CAU44	227,229,231	225	–
CAU57	201,203,215,217	205	221
CAU64	167,169,171,173,175,181,183	159	161
CAU65	177,179,181,183,185,187	–	191
CAU68	263,265,267,269,271	–	–
CAU69	98,100,106,108,110	112	104
CAU75	182,184,194,198,200,204,206	196	–
CAU76	224,226,228,230,236	218,222,232	242,246,248,252
CAU78	117,119	121	–
CAU84	202,204,206,208,210,212	200	–
CAU85	244,246,248,266,268,272	226,236,262,264	228,230,252,270,274
CAU97	150,154,158,162	152	160,164
CAU98	162,164,166,168,170	160,178	172,174

The relationship between microsatellite marker alleles from the Rhode Island Red and Green-legged Partrigenous hens and egg production and quality traits in mapping population was studied by Wardęcka et al. [29]. Polymorphism of 23 microsatellite markers was investigated and 30 traits of egg production and quality measured during the laying period. The results confirmed that the analyzed microsatellite *loci* may be linked to the genes affecting egg production and quality traits. In turn, Chatterjee et al. [1] studied the microsatellite variability and its relationship to the other egg production traits in the chicken. Nine microsatellite markers were explored. Three of the studied microsatellite *loci* were found significantly (*p* < 0.05) related to egg production traits.

The results of this study indicate that between the groups analyzed, the LP hens showed significantly more specific alleles (56.0 % of the total pool of specific alleles), whereas in HP hens specific alleles consisted of 43.9 % of the total pool of these alleles.

The results of the current investigation show the usefulness of microsatellite *loci* as polymorphic genetic markers of laying performance of ostriches as well as possible association of particular allele to egg production. Identification of such markers performed for the first time in the ostrich may be useful in ostrich breeding as a new tool in further genetic improvement of ostrich flocks.
